# Prevalence of hypertension in Iran: An updated systematic review and meta-analysis of community-based studies 

**DOI:** 10.22088/cjim.14.43.607

**Published:** 2023

**Authors:** Salman Mohammadi, Soheil Hassanipour, Hamed Delam, Hossein-Ali Nikbakht, Zohreh Ghaem Far, Donya Firoozi, Elnaz Ghaem Far, Hamid Abdolazimi, Ali Ghaffarian-Bahraman

**Affiliations:** 1 Department of Nutrition, School of Health and Nutrition, Lorestan University of Medical Sciences, Khorramabad, Iran; 2 Gastrointestinal and Liver Diseases Research Center, Guilan University of Medical Sciences, Rasht, Iran; 3 Student Research Committee, Larestan University of Medical Sciences, Larestan, Iran; 4 Social Determinants of Health Research Center, Health Research Institute, Department of Biostatistics and Epidemiology, Faculty of Public Health, Babol University of Medical Sciences, Babol, Iran; 5 Department of Clinical Nutrition, School of Nutrition and Food Sciences, Shiraz University of Medical Sciences, Shiraz, Iran; 6 Department of Clinical Nutrition, South Tehran Branch, Islamic Azad University, Tehran, Iran; 7 Occupational Environment Research Center, Rafsanjan University of Medical Sciences, Rafsanjan, Iran

**Keywords:** Hypertension, ACC/AHA guidelines, Iran

## Abstract

**Background:**

Hypertension (HTN) is one of the primary risk factors for heart disease and stroke worldwide. The present meta-analysis was aimed to systematically review and statistically estimate the prevalence rate of pre-hypertension (PHTN) and HTN in the Iranian child/adolescent and adult age groups.

**Methods:**

In this study, four International databases, including PubMed, Scopus, Web of Science, and Cochrane, as well as three Iranian databases, including SID, Magiran, and IranMedex, were separately investigated for articles published before January 2021. Also, we estimated the pooled effect size for the prevalence of PHTN and HTN in children/adolescent and adult age groups. Stata software (version 14.0) was used for all statistical analyses.

**Results:**

From a total of 1185 articles found in database searches, fifty-one were included in the meta-analysis. The prevalence of HTN in the Iranian adult population was 26.26% (25.11 % and 26.22 % for women and men, respectively). Meanwhile, the prevalence of PHTN and HTN in the child/adolescent age group was 8.97% (95% CI 7.33 - 10.61) and 8.98% (95% CI 7.59 - 10.36), respectively.

**Conclusions:**

This study provides information which can be used for various purposes, including study designing. Further nationwide surveys should be carried out to obtain accurate information on the HTN prevalence rate, particularly based on the American College of Cardiology /American Heart Association guidelines in the Iranian population.

Hypertension (HTN), a common chronic disease that is becoming an epidemic globally, coincides with the increasing prevalence of obesity, metabolic syndrome, and type 2 diabetes (1-3). The risk factors for hypertension include unhealthy diets, physical inactivity, consumption of tobacco and alcohol, being overweight, family history, age over 65 years and exposure to environmental pollutants (4-6). It is well-established that uncontrolled HTN may result in health consequences, including stroke and heart failure, which are two leading causes of mortality worldwide. According to WHO, the HTN affects about one billion adults worldwide, with more than 9 million deaths annually (7). Accumulated research has indicated that correction of high blood pressure can significantly alleviate cardiovascular complications and mortality (8, 9). The strong association of HTN with a wide range of chronic diseases, from metabolic syndrome and obesity to cardiovascular disease and stroke, underscores the need for a precise definition of the disease. The 2017 American College of Cardiology (ACC)/American Heart Association (AHA) hypertension guidelines, change the definition of HTN from 140/90 mm Hg to 130/80 mm Hg for systolic/diastolic blood pressure (SBP/DBP), surprisingly (10). 

Although the definition of normal BP has remained the same as the Seventh Report of the Joint National Committee (JNC7), i.e., SBP <120 mmHg and DBP <80 mmHg, the new guidelines has divided the “pre-hypertension” phase into two stages of “raised blood pressure” (i.e., SBP of 120 to 129 mmHg with DBP <80 mmHg) and "stage 1 hypertension" (i.e., SBP of 130 to 139 mmHg or DBP of 80 to 89 mmHg). Also, Stage 2 HTN is described in 2017 ACC/AHA guidelines as SBP/DBP of at least 140/90 mmHg, rather than the values of at least 160/100 mmHg in JNC7 (11).

These fundamental changes have been created mainly due to the double-fold risk of cardiovascular disease in adults with HTN (12). According to 2017 ACC/AHA hypertension guidelines, more than 70 million individuals in the US (63% of the population) and 266.9 million individuals in China (55% of the population) at the age group of 45-75 years are afflicted by HTN. This indicates an increase in the HTN prevalence of 45.1% and 26.8% in China and the US, respectively (13). Furthermore, adaptation to the 2017 ACC/AHA guidelines in Canada resulted in an 8.7% increase in the adult-age HTN prevalence (14). These findings highlight the importance of an updated estimation of the HTN prevalence based on new classifications in Iran. Two previous systematic reviews in the Iranian population had used JNC7 guidelines, according to which the prevalence of HTN was 23% and 50% in adults aged 30-55 years and above 55 years, respectively (15, 16). 

Moreover, an extensive national survey indicated that approximately 43% of Iranian adults suffer from high blood pressure based on JNC7 guidelines (17). According to a previous meta-analysis, the overall prevalence of PHTN and HTN in Iranian adult population estimated to be 31.6% and 20.4%, respectively (18).

Giving the definite economic impacts of HTN on healthcare systems as a significant risk factor for cardiovascular diseases (CVD) and stroke, an accurate estimation of its prevalence rate seems necessary worldwide (18). Noteworthy, four years after the release of the new ACC/AHA guidelines, many Iranian medical centers still follow JNC7 instructions for the diagnosis and control of high blood pressure. The present study was aimed to systematically review and statistically estimate the prevalence rate of hypertensive disorders in the child/adolescent and adult age groups by assessing the available community-based reports in Iran.

## Methods

**Data source and searches: **The present systematic review and meta-analysis study was designed and implemented in 2021 according to the preferred reporting items for systematic reviews and meta-analysis (PRISMA) guidelines (19, 20). Four International databases, including PubMed, Scopus, Web of Science, and Cochrane, as well as three Iranian databases, including SID, Magiran, and IranMedex, were separately investigated for articles published before January 2021. Google Scholar was also searched for grey literature. Additionally, reference lists of included articles were screened to find related titles.

Following keywords were used in the internal and the international database searches: (“Hypertension” OR “HTN” OR “Prehypertension” OR “PHTN” OR “Blood Pressure” OR “Arterial Pressure” OR “systolic” OR “Diastolic” OR “non-communicable disease” OR “cardiovascular diseases” OR “CVD” OR “metabolic syndrome” OR “Cardio-metabolic”) AND (“Prevalence” OR “Epidemiology” OR “Occurrence”) AND “Iran”. All found studies were exported into Endnote X8, and duplicate titles were automatically omitted. Then, related articles were identified through screening the subjects, abstracts, and full texts by two independent researchers. Finally, associated articles were thoroughly studied for data extraction. 

**Definitions: **Given the different updates of guidelines, the included articles had various definitions for hypertension depending on their publication year. The PHTN definition ranged from SBP = 120-139 mmHg to 130-139 mmHg and, or DBP = 80-85 mmHg to ≥ 90 mmHg in adult population. Also, its definition in children/adolescent age group ranged from SBP/DBP values between 90^th^ and 95^th^ percentiles to the values ≥ 90^th^ percentile of blood pressure. In addition, definitions were also various for HTN ranging from the SBP/DBP values ≥ 122/82 mmHg to the values ≥ 140/90 mmHg in the adult population. In the children/adolescent age group, the HTN definitions were varied from blood pressure values ≥ 90^th^ percentile to the values ≥ 95^th^ percentile.

These heterogeneities were not only specific to the blood pressure definitions but also included the target population age groups. Therefore, we categorized the included studies into two main subgroups based on the age category of their target population, namely the children/adolescent (age 6-20 years) and the adult (age ≥ 15 years) age groups.

**Inclusion and exclusion criteria: **All articles reporting the HTN and the PHTN prevalence rate in the Iranian population were included in the present study. Accessibility to full texts, publication date January 2021, and writing language of English or Persian were other inclusion criteria. On the contrary, review and meta-analysis studies were omitted after reference list screening. 

**Quality of the studies: **Two reviewers screened and assessed the relevancy of the studies separately, mainly according to papers' titles and abstracts, as well as full-text investigation in a few cases. The final decision was made on subject selection discussing any disagreement between the reviewers. Joanna Briggs Institute (JBI) critical appraisal checklist was used to assess the quality of articles (21). 

**Data extraction: **All eligible articles were thoroughly reviewed for data extraction using a previously-prepared checklist. The following information was extracted: first authors' name, publication year, sample size, location of the study, age group and gender of the target population, prevalence rate and 95% confidence intervals for both PHTN, and HTN. 

**Statistical analysis: **Forest plots for estimation of pooled effect sizes were used based on a random-effects model. Between-study, heterogeneity was investigated using Cochran Q-test (p-value <0.1 as significant) and I-squared index$\left(I^{2}\right)$. In the event of significant between-study heterogeneity, meta-regression and subgroup analyses were conducted. Furthermore, the possibility of publication bias was assessed using both visual and quantitative methods (funnel plot and Egger’s regression test, respectively). Stata software (Version 14.0, Stata Corp, College Station, TX, USA) was used for all statistical analyses.

## Results

**Figure 1:**
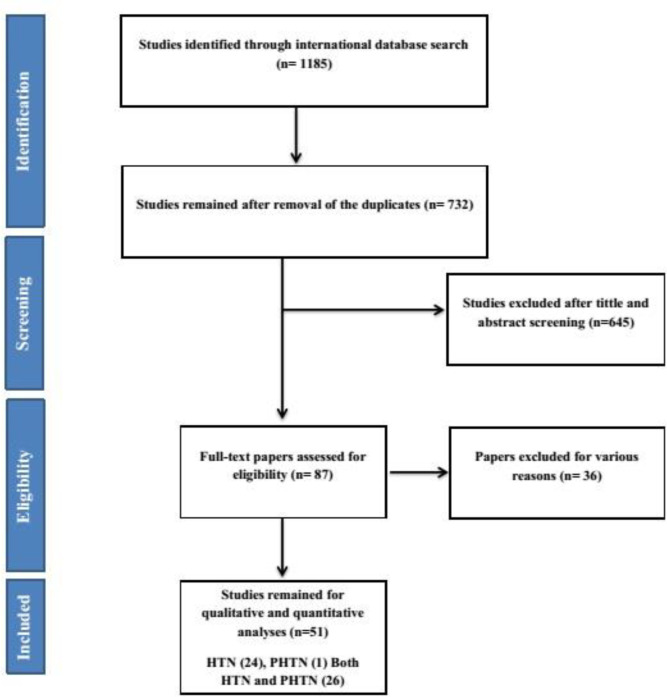
Flowchart of study selection

**Study selection: **Searching the international databases, a total of 1185 articles were found. After omitting duplications, 732 papers were remained for the relevancy assessment. Finally, 51 articles were considered eligible, and included in the final analysis. The selection process of the included studies is presented in figure 1. 

**Description of included studies: **Among the included studies, one paper reported the prevalence of PHTN (22), 24 articles reported the HTN prevalence (23-46) and the rest had information associated with both the HTN, and the PHTN prevalence rate (47-72). Furthermore, we analyzed 30 articles in adult and 21 articles in child/adolescent age groups. Noteworthy, one study specifically had reported the prevalence values in the elderly population, which was excluded. The basic characteristics of included studies are shown in table 1.

**Heterogeneity: **Results of I^2 ^index and chi-square test demonstrated significant heterogeneities between studies even in subgroup investigations. As a result, the random effects model was applied in all analyses. The heterogeneity-test results are presented in table 2.

**Meta-analysis: **According to this study, the HTN prevalence rates were 26.26% (95% CI, 22.36 – 30.17) and 8.98% (95% CI, 7.59 – 10.36) in the adult (Sup1 – Sup3) and the children/adolescent age groups (Sup 4 – Sup 6), respectively. Meanwhile, the pooled prevalence rate of PHTN was 37.31% (95% CI, 31.11- 43.5) in adults (Sup 7 – Sup 9) and 8.97% (95% CI, 7.33-10.61) in the children/adolescent age group (Sup10 - Sup12). The results of subgroup meta-analysis based on different age and sex categories are shown in detail in table 2.

**Figure 2:**
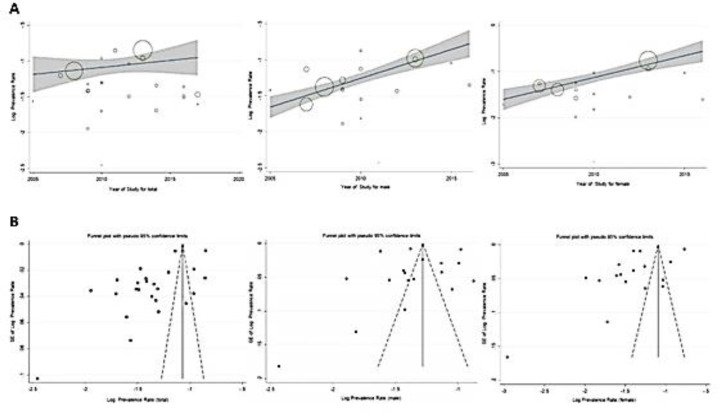
Results of meta-regression for hypertension prevalence rate based on publication year of studies (A), and Funnel plots of standard errors for publication bias assessment (B)

**Meta-regression and Publication bias: **Meta-regression analysis showed no significant relationship between the PHTN/HTN prevalence rates and the publication-year of included studies. Although the results were not statistically significant, blood pressure in both adult and children/adolescents age groups raised by approximately 3% per year of age increase (figure 2-A). Furthermore, the results of Egger's regression test indicated a significant publication bias for included studies (P<0.001), confirmed by the visual test of funnel plots (Figure 2-B). However, there was no significant publication bias when studies were analyzed in subgroups based on gender (p-value of 0.655 and 0.052 for men and women, respectively).

**Table 1:** Basic information of included studies

**First Author**	**Study** **Location**	**Prevalence Outcome**	**Sample Size (n)**	**Age group (Year)**	**Gender**
**Abdoolahi**	Golestan	Both	5000	17-70	Both
**Aghaei Meybodi**	TBT *	Both	3049	20-64	Both
**Ashrafi**	Tehran	HTN	10288	6-13	Both
**Ataei**	Tehran	HTN	6038	13-18	Both
**Azimi Nezhad**	Khorasan	Both	4519	15-65	Both
**Azizi**	Kermanshah	Both	4718	> 15	Both
**Badeli**	Rasht	Both	2072	7-17	Both
**Basiratnia**	Shiraz	HTN	2000	11-17	Both
**Baskabadi**	Mashhad	HTN	704	> 18	Both
**Ebrahimi**	Mashhad	Both	9762	30-65	Both
**Esteghamati**	Iran	Both	68250	25-64	Both
**Esteghamati**	Iran	Both	4233	25-64	Both
**Esteghamati**	Iran	Both	8218	25 -70	Both
**Falah**	Tehran	HTN	8848	7-11	Both
**Fallah**	Iran	HTN	13486	6-18	Both
**Ghanbarian**	Tehran	HTN	2575	10-17	Both
**Ghorbani**	Semnan	HTN	3799	30-69	Both
**Hakim**	Ahvaz	Both	1100	6-12	Both
**Heydari**	Shiraz	Both	341	20-54	Male
**Janghorbani**	Iran	Both	69722	25-64	Both
**Janghorbani**	Iran	Both	89404	15-65	Both
**Kalani**	Yazd	Both	1130	> 18	Both
**Kalani**	Yazd	PHTN	456	> 18	Male
**Kassaei**	Zanjan	Both	997	15-67	Both
**Kazemi**	Birjand	HTN	1286	15–70	Both
**Kelishadi**	Iran	HTN	21111	6-18	Both
**Kelishadi**	Iran	HTN	5682	10-18	Both
**Khajedaluee**	Mashhad	HTN	2974	16-90	Both
**khosravi**	Shahroud	Both	5190	40–64	Both
**Khosropanah**	Shiraz	Both	3115	21-73	Both
**Malekzadeh**	Golestan	HTN	50045	40-75	Both
**Mehr-Alizadeh**	Semnan	HTN	2125	9-17	Both
**Mehrdad**	Tehran	HTN	1067	3-9	Both
**Mehrkash**	Gorgan	HTN	450	15-17	Both
**Mirzaeipour**	Kerman	HTN	803	14-17	Both
**Mohammadi**	Ilam	Both	1075	7-11	Both
**Mohkam**	Tehran	HTN	425	7-11	Both
**Moravej**	Ahvaz	Both	1707	10-17	Both
**Motiei-langarodi**	Qazvin	HTN	5917	7-12	Both
**Najafipour**	Kerman	Both	5858	15-75	Both
**Namayandeh**	Yazd	HTN	2000	20-74	Both
**Peymani**	Fars	HTN	3916	15-64	Both
**Rafraf**	Tabriz	Both	985	14-17	Female
**Rahmanian**	Jahrom	Both	892	≥30	Both
**Sahebi**	Shiraz	Both	1027	>19	Both
**Salem**	Rafsanjan	Both	1221	11-17	Female
**Shahraki**	Zahedan	HTN	2300	≥30	Both
**Shidfar**	Tehran	HTN	1184	10-13	Both
**Shojaei**	Jahrom	HTN	405	≥30	Male
**Tabrizi**	East Azerbaijan	Both	2818	15–64	Both
**Zardast**	Birjand	Both	1521	6-11	Both

N (total number of subjects); PHTN (pre-hypertension); HTN (Hypertension) * both (PHTN and HTN)

TBT (Tehran - Booshehr - Tabriz)

**Table 2:** Meta-analysis and heterogeneity results for the prevalence rates of hypertension and pre-hypertension among the Iranian children/adolescent and adult age groups

	**Age group**	**Sex**	**Prevalence Rate (%)**	**I** ^2^	**P-value**
**HTN**	Adults	Male	26.22 (22.79 – 29.66)	99.3	<0.001
		Female	25.11 (19.82 – 30.39)	99.7	
		Overall	26.26 (22.36 – 30.17)	99.7	
	Children/ Adolescents	Male	9.06 (6.40 – 11.71)	97.6	<0.001
		Female	8.19 (5.90 – 10.47)	98.0	
		Overall	8.98 (7.59 – 10.36)	98.6	
**PHTN**	Adults	Male	40.73 (33.53 – 47.93)	99.7	<0.001
		Female	32.62 (27.20 – 38.04)	99.6	
		Overall	37.31 (31.11 – 43.52)	99.9	
	Children/ Adolescents	Male	9.13 (6.13 – 12.13)	82.5	0.017
		Female	8.41 (2.56 – 14.26)	98.1	<0.001
		Overall	8.97 (7.33 – 10.61)	84.2	

PHTN (pre-hypertension); HTN (Hypertension) Results of the meta-analysis and heterogeneity for prevalence rates of HTN and PHTN among Iranian children/adolescents and adults based on Joint National Committee (JNC7) guidelines.

## Discussion

The HTN as a public health problem has become a major cause of concern worldwide (73). By 2025, it has been projected that 75% of hypertensive individuals will be residing in developing countries (74). Recent ACC/AHA guidelines have defined new categories for HTN in the adult population by tightening cut-offs. As a result of this approach, prompt and significant growth has occurred in the prevalence of HTN in different populations. The majority of patients with PHTN will be labeled as hypertensive based on the new definitions. 

Although it has been a considerable time since the publication of the ACC/AHA guidelines, most Iranian studies still use the JNC7 guidelines for the diagnosis of HTN. On the other hand, the gaps and overlaps between the cut-offs used in previous reports prevent the conduction of a comprehensive meta-analysis to estimate the pooled prevalence of HTN according to the 2017 ACC/AHA guidelines. Given the prevalence rate of PHTN and HTN estimated in the present study, it seems that the prevalence of HTN will increase to more than 50% in the Iranian population if the 2017 ACC/AHA guidelines are used as a basis for diagnosis. In line with our estimation, some other studies indicated considerable increases in HTN prevalence rates in Iran due to the introduction of ACC/AHA guidelines. A 2011 study in a population of 10,000 in Iran showed that the prevalence of HTN was 27.6% and 25.8% among adult males and females, respectively (75). In 2019, the researchers reconsidered information for the same study based on new guidelines and reported that overall HTN prevalence rate rose to 48.2% in adults, 44.3% in women, and 52% in men (76). Moreover, re-analyzing data from another Iranian study with a relatively similar population in Tehran also showed that the percentage of hypertensive patients who did not receive blood pressure control medications increased from 12.6% based on the JNC7 to 42.7% based on new guidelines. These values were 20.4% and 47.1% for patients receiving anti-hypertensive drugs, respectively (77).

Similar alterations have been reported in some other countries. The prevalence rate of HTN rose from 31.9% based on the JNC7 guideline to 45.6% according to the 2017 ACC/AHA among American adults. These guideline changes led to a 31.1 million increase in the population of hypertensive adults and 4.2 million in the number of individuals requiring antihypertensive medications (78). This challenge was more pronounced in the middle-aged and elderly population. According to a US study, indicated that the prevalence of HTN rose from 26.8% to 63% in the 45-year to the 75-year population after considering the 2017 ACC/AHA definitions (13). 

Another study carried out on a population of postmenopausal rural women aging 40–70 years in Bangladesh indicated that the prevalence of HTN was 67.5% based on the new guidelines (79). Furthermore, it is reported that the introduction of 2017 ACC/AHA guidelines has resulted in prompt rises in HTN prevalence from 25% to 50% in China (17), from 36% to 58% in Japan (80), from 21.2% to 44.2% in Nepal (81), and from 13.1% to 40.1% in India (82).

Different countries around the world have faced many challenges due to this new definitions. Increasing the population of hypertensive patients has resulted in considerable growth in demand for antihypertensive medications, and a consequent dramatic increase in the proportion of related economic burdens on the health systems. On the other hand, controlling blood pressure complications in their early stages by lifestyle modification and drug treatment can more properly prevent HTN -induced diseases including heart failure, myocardial infarction, brain damage, and kidney failure (83, 84).

However, some experts hold the opposite points of view and believe that industry cherishes expanding the disease definition to label more individuals in need of medical treatment. Although these guidelines reinforce the message that inexpensive medications including thiazides are among the most appropriate choices; many patients will need combinations of expensive drugs to attain the lower target of blood pressure (85). Furthermore, the likelihood of the adverse events' incidence will increase with expanded treatment (86). Revision in HTN definition has brought about changes in prevalence and survival rates of related disorders. The findings of the recently published study have indicated that the proportion of HTN-induced stroke survivors in the United States which was 29.9% according to JNC7, rose to 49.8% exerting new guidelines (87). These findings are in accordance with the purpose outlined in the 2017 ACC/AHA strict guidelines for HTN risk reduction before consequent complications such as myocardial infarction and stroke (10). Nevertheless, lifestyle modification is in greater emphasis to decline the necessity of pharmacological interventions (78).

There were limitations in our study to be taken into consideration. Differences in blood pressure cutoffs used for HTN diagnosis made it challenging to conduct a single meta-analysis on the extracted data. However, we classified the studies into two categories to minimize the overlaps and gaps of the cut-offs. Furthermore, subgroup analyses were not conducted based on the major blood pressure affecting factors including body mass index, physical activity, smoking, and alcohol consumption due to insufficient data. However, we applied all meta-analyses in a random-effects model to nullify the impacts of heterogeneities on pooled estimation. Despite these limitations, our study had some advantages.

 We extracted all national and local information to make a relatively precise estimation, and meta-regression analyses were conducted to identify heterogeneity sources. 

Our estimate of the prevalence of hypertensive disorders is concerning in Iran. It seems that the number of patients with HTN will drastically increase in Iran exerting the 2017 ACC/AHA guidelines. Although the findings of the current study can be used for various purposes, further nationwide surveys should be carried out using the 2017 ACC/AHA guidelines to provide more accurate information on the HTN prevalence rate in the Iranian population.
